# Advanced Nasal Septal Squamous Cell Carcinoma: Legal Implications of Treatment Delay Due to COVID-19

**DOI:** 10.7759/cureus.92839

**Published:** 2025-09-21

**Authors:** Shivani Raizada, Sherrie Wang, Vlad Kushnir, Yekaterina Koshkareva

**Affiliations:** 1 Otolaryngology - Head and Neck Surgery, Cooper Medical School of Rowan University, Camden, USA; 2 Otolaryngology - Head and Neck Surgery, Emory University School of Medicine, Atlanta, USA; 3 Law, VB Kushnir, LLC, Trevose, USA; 4 Otolaryngology - Head and Neck Surgery, Cooper University Healthcare, Camden, USA

**Keywords:** covid-19, head and neck, malpractice allegations, otolaryngology-head & neck surgeons, squamous cell carcinoma

## Abstract

During the COVID-19 pandemic, there were state-mandated suspensions of elective surgeries, which raised legal concerns about delays in treatment. Skin squamous cell carcinoma (SCC) is one condition where postponement of care can lead to aggressive progression and significant morbidity. This case explores the legal and clinical implications of surgical delays during the pandemic. A 93-year-old male presented with advanced nasal septal SCC two weeks after New Jersey lifted its suspension on elective procedures. The lesion, which began as a small growth nearly a year earlier, had rapidly progressed during the suspension, rendering it unsuitable for Mohs micrographic surgery. Otolaryngology proceeded with the case due to extensive invasion evident on imaging. The patient required a partial rhinectomy and complex reconstruction, which resulted in an uneventful recovery with minimal complications. Medical malpractice claims require a breach of duty, injury, and causation. However, during a public health emergency, crisis standards of care may redefine the physician’s duty. Guidelines from the Institute of Medicine and the American College of Surgeons (ACS) supported triaging surgeries based on urgency and survivability, providing a legal and ethical framework for such decisions. In this case, the surgical delay was aligned with ACS recommendations for elective cancer surgery, likely protecting the providers from legal liability. This case demonstrates the importance of clearly defined crisis standards of care in protecting healthcare providers during emergencies. The multidisciplinary response following the delay ensured optimal patient outcomes and prevented legal consequences, highlighting the value of adaptive guidelines and expert consensus during public health crises.

## Introduction

COVID-19 brought unprecedented circumstances, including forced cancellations and delays of various surgeries to free up space for those sick with the virus and minimize risk to the providers [1]. As the state of New Jersey began to enforce vague yet stringent mandates instructing delays on surgeries deemed “elective,” medical providers expressed concerns regarding potential legal liability that would inevitably arise [1,2].

Regarding the disease progression of skin squamous cell carcinomas (SCC), treatment delay between lesion presentation and Mohs micrographic surgery can result in significant growth, local epidermal and bony destruction, and metastasis [3]. Therefore, as physicians navigate novel obstacles to surgical intervention, cases such as the one presented here must be reviewed to explore the potential for legal ramifications of COVID-19 pandemic treatment delay.

## Case presentation

Two weeks after the New Jersey (NJ) Executive Order No. 109 suspension of elective surgeries was lifted, a 93-year-old male presented to the Cooper University Hospital Dermatology Office for the evaluation of nasal septal SCC. It started as a small lesion almost one year prior. The lesion rapidly progressed within the following few months after biopsy, as the patient was unable to schedule a Mohs micrographic surgery consultation due to the COVID-19 state-mandated suspension of all elective surgeries. Following resolution of the suspension, dermatologic consultation revealed progression to advanced nasal septal SCC, which was no longer amenable to Mohs procedure, and the patient was referred to an Otolaryngology Head and Neck Cancer specialist. Written informed consent was obtained from the patient for publication of this case report and the following de-identified images. Identifying features have been obscured to protect the patient’s identity.

**Figure 1:**
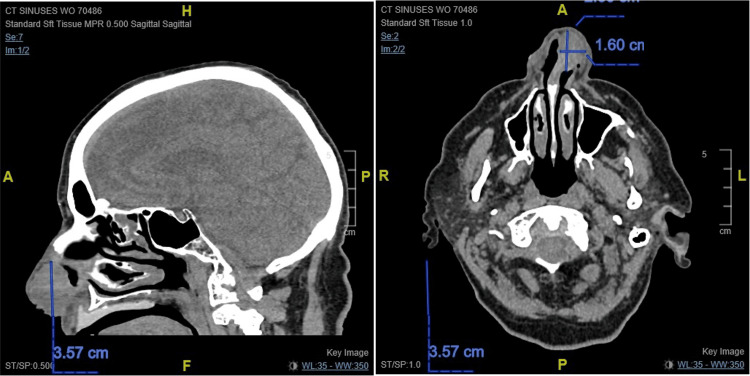
Sagittal (left) and axial (right) CT showing a 2.6 × 1.6 × 3.6 cm mass occupying the left anterior nasal cavity and eroding through the cartilaginous septum to the contralateral nasal cavity.

**Figure 2:**
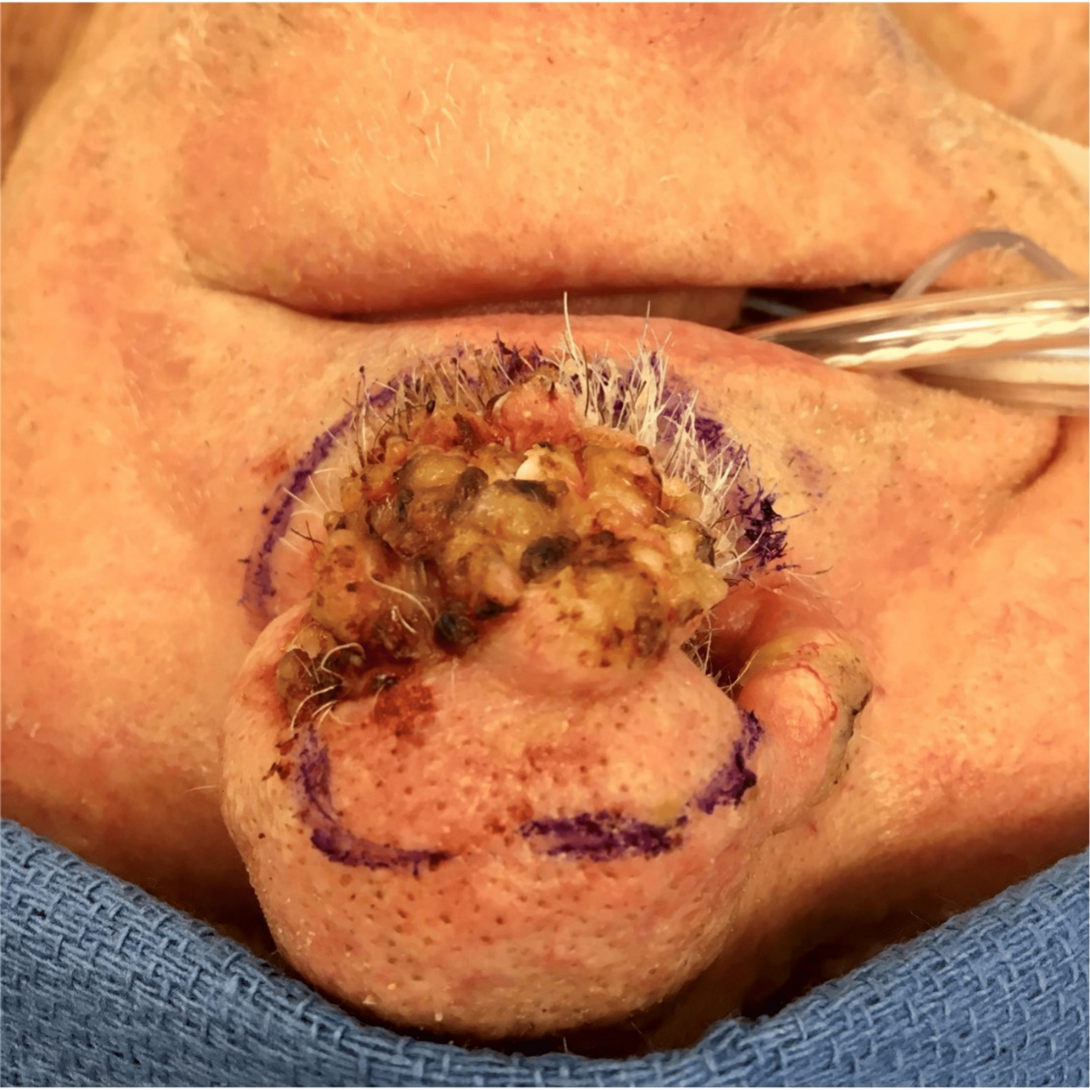
Lesion prior to surgery.

**Figure 3:**
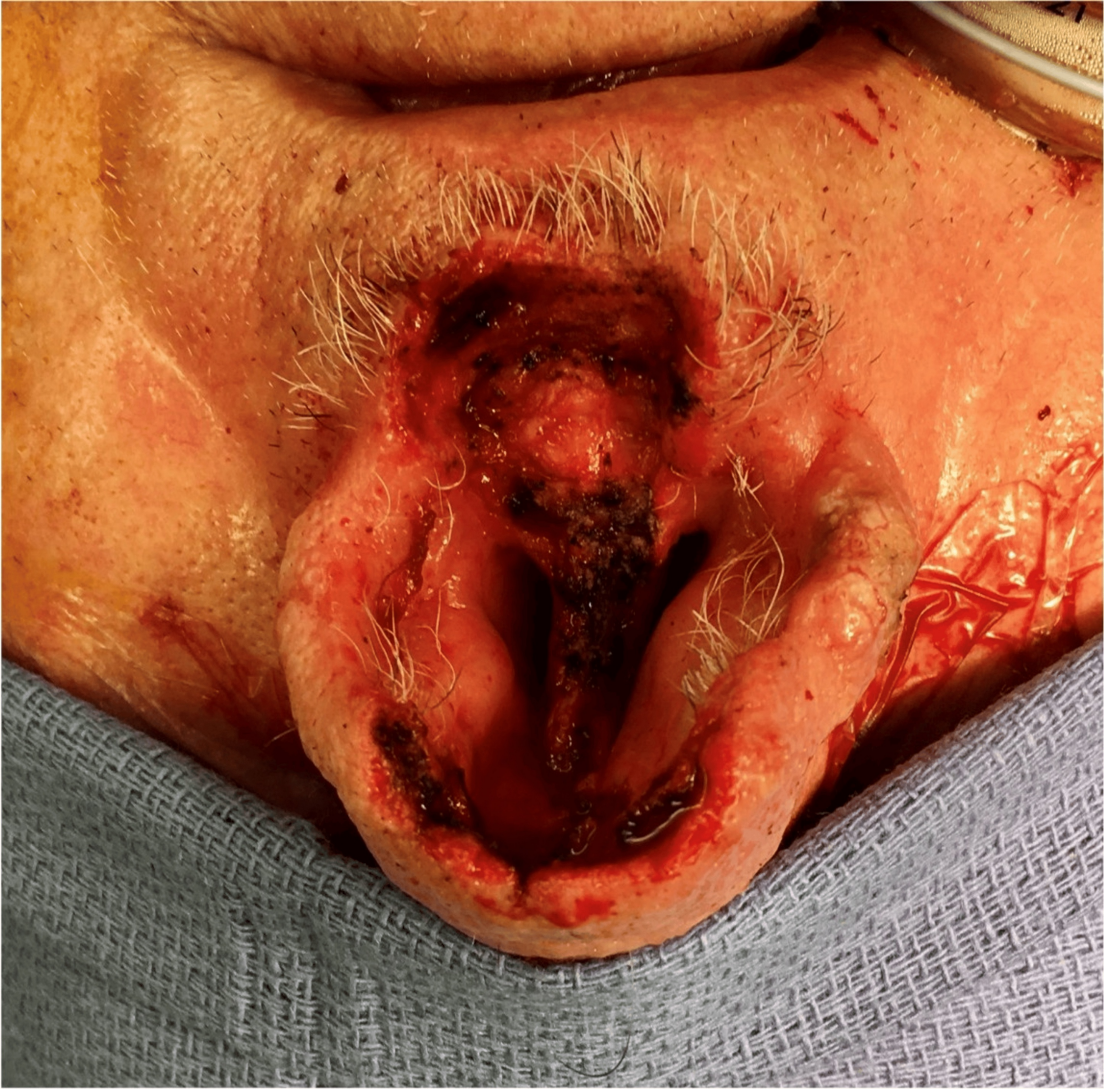
Surgical defect after partial rhinectomy.

**Figure 4:**
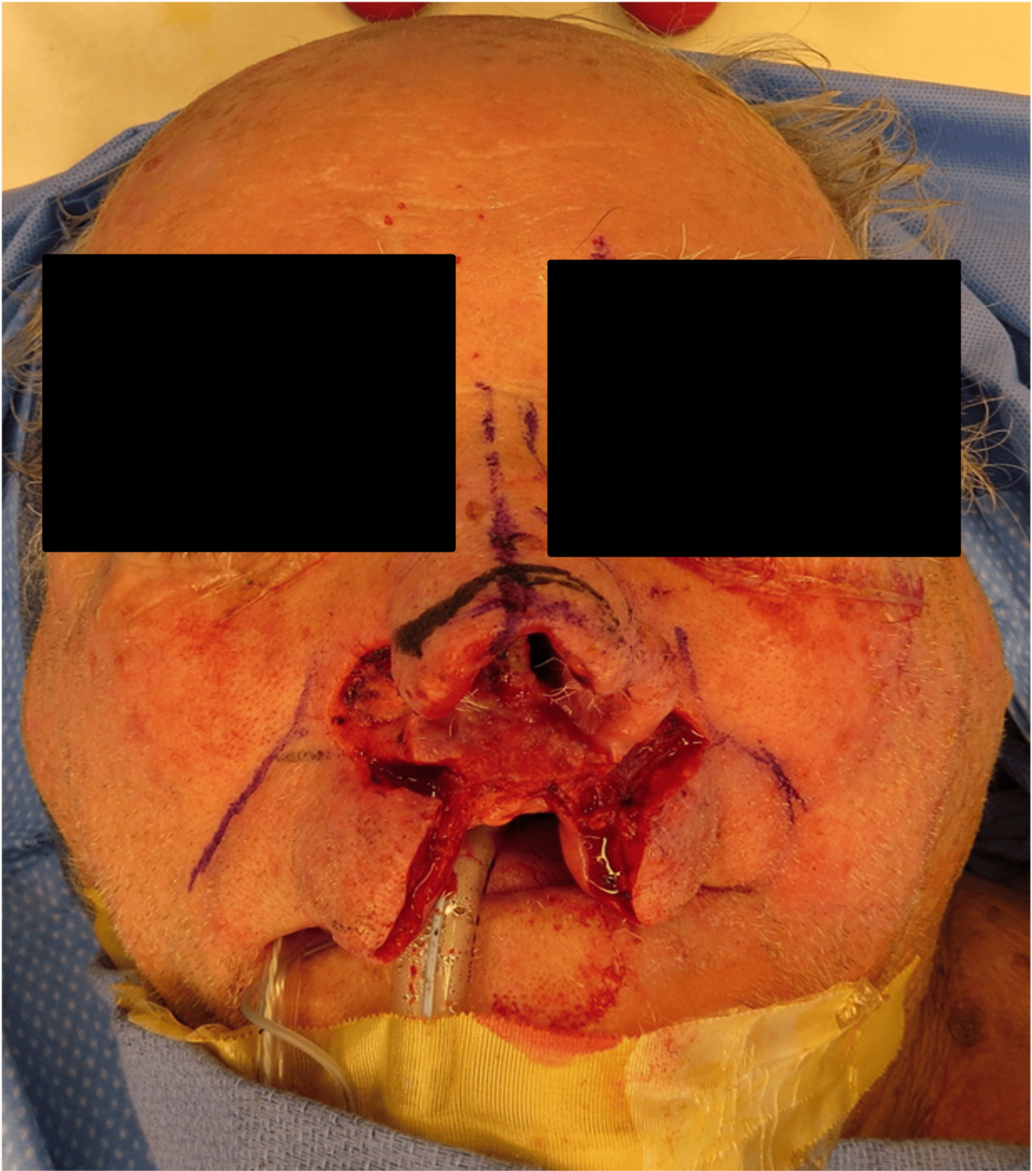
Upper lip advancement.

**Figure 5:**
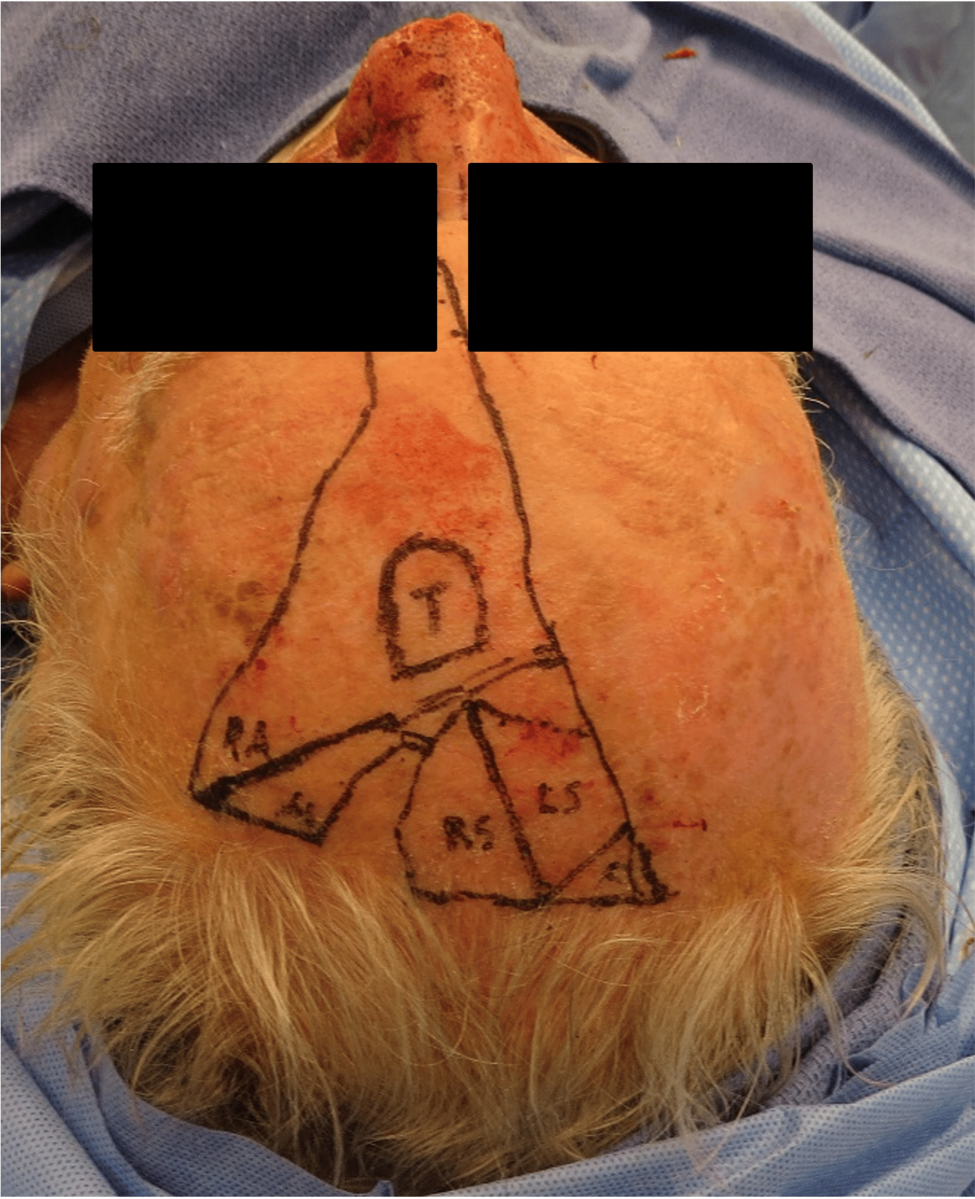
Extended forehead flap.

**Figure 6:**
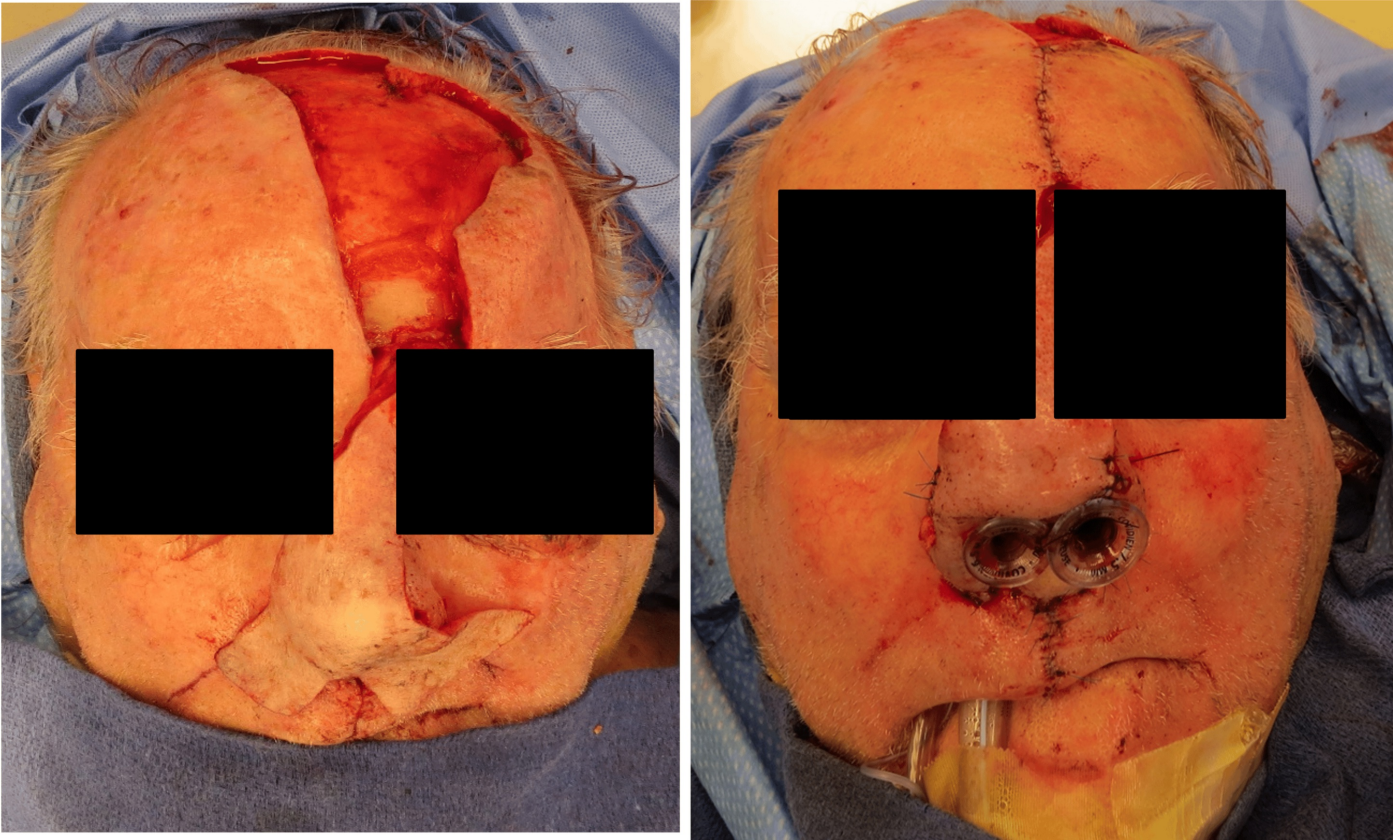
Flap inset.

CT of the face and neck revealed a left septum soft tissue mass occupying the ipsilateral anterior nasal cavity, eroding through the cartilaginous septum to the contralateral naris (Figure 1). The patient underwent a partial rhinectomy, including the majority of the anterior septum and nasal tip, and a partial resection of the superior columella down to the maxilla (Figures 2-4). The defect necessitated a multi-stage forehead flap and bilateral upper lip advancement flaps for reconstruction (Figures 5, 6).

**Figure 7:**
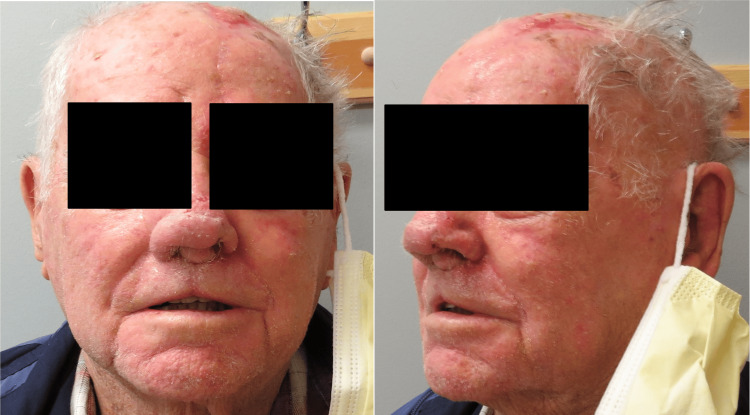
Two weeks postoperatively.

**Figure 8:**
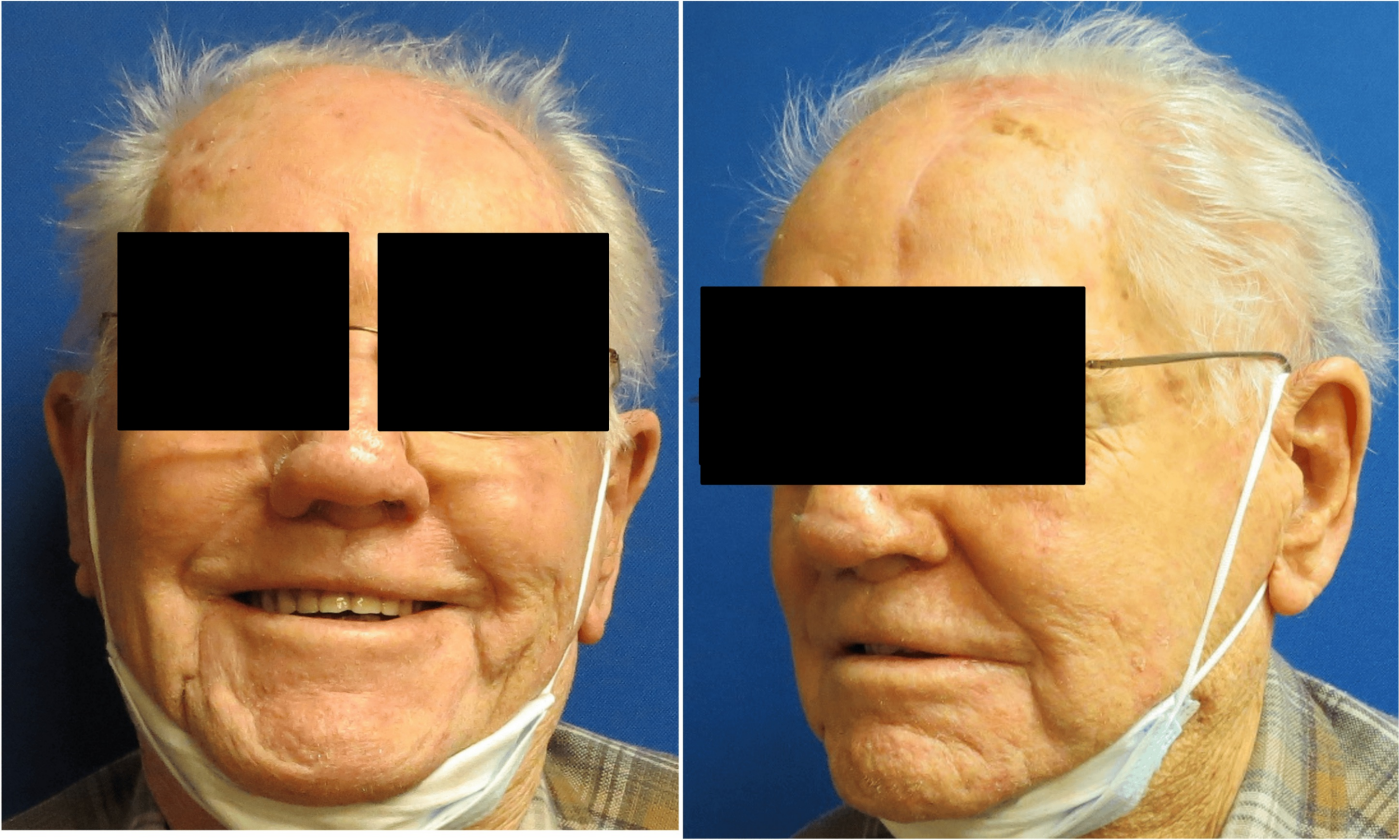
Three months postoperatively.

Minimal side effects, including rhinorrhea and right nostril swelling, were noted; however, the patient was able to breathe through the nose. The patient had no complications on subsequent follow-up visits (Figures 7, 8).

## Discussion

In the United States, medical malpractice laws are governed by the individual states. To establish a medical malpractice lawsuit, a plaintiff (patient) must establish certain elements. First, the plaintiff must show that the defendant (physician) owed a legal duty to the plaintiff [4]. In most cases, legal duty is easily established because there is scant opposition that an agreement to provide medical care was formed at the establishment of a physician-patient relationship [4]. Second, the plaintiff must establish that the defendant breached his/her duty by deviating from the standard of care [4]. Third, the plaintiff must show that he/she suffered injuries or other damages [4]. Fourth, the plaintiff must show that his/her injuries and damages were proximately caused by the defendant’s breach of duty (i.e., by the defendant’s deviation from the applicable standard of care) [4].

**Figure 9:**
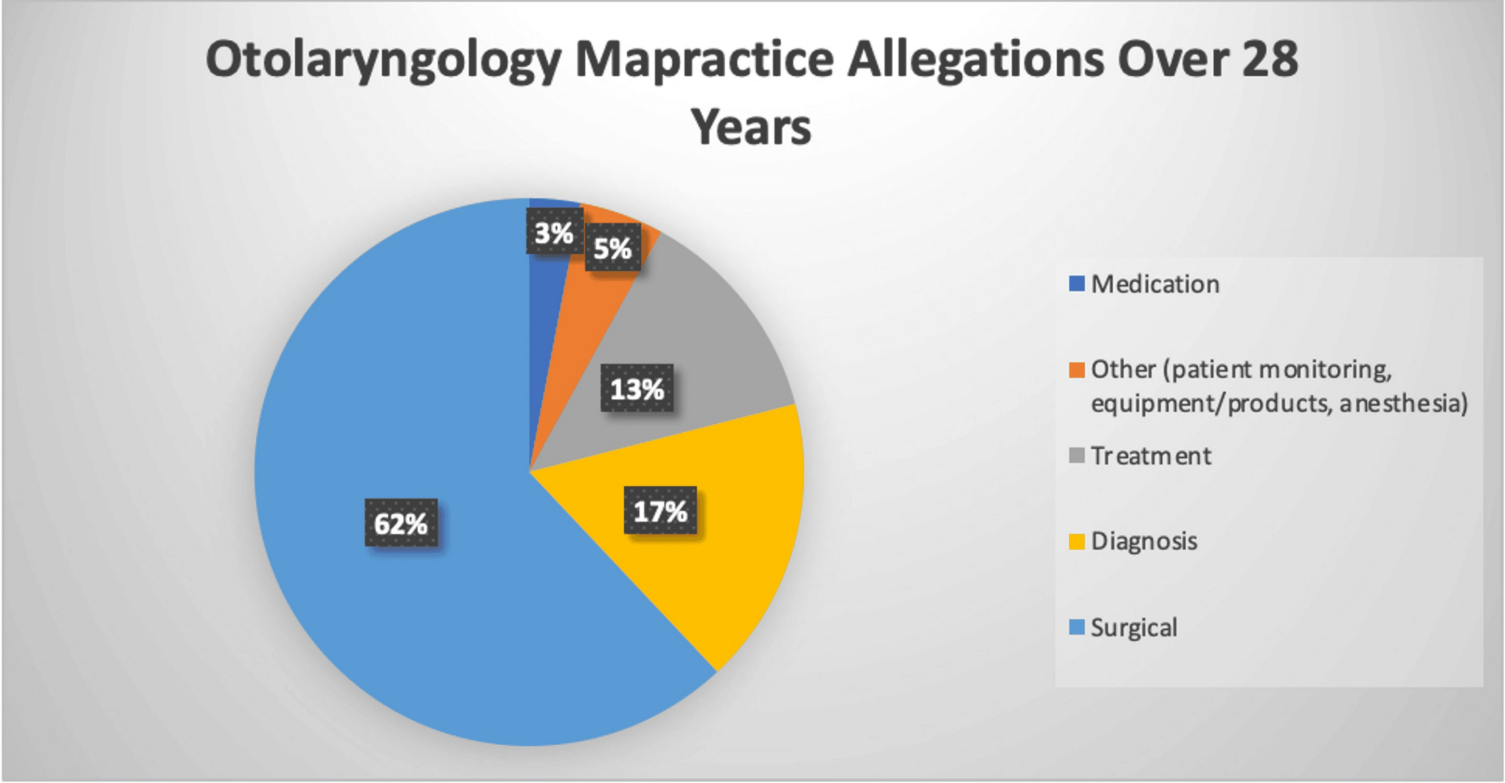
Otolaryngology malpractice allegations over 28 years: a pie chart depicting the proportion of each malpractice allegation type filed against U.S. otolaryngologists between 1991 and 2018.

Figure 9 depicts U.S. Otolaryngologic malpractice allegations made between 1991 and 2018, categorized by allegation type, and includes proportions for each [5]. The law concerning medical malpractice arising from COVID-19-related treatment delays is yet to be fully developed, as most such cases are laboriously being processed through the legal system. Most cases are situationally specific and fact-dependent, making it nearly impossible to outline a formula for legal outcomes. However, it can be assumed that most COVID-19-related treatment delay cases will teeter on the “breach of duty” element, wherein the plaintiff would have to present expert witness testimony that the decision to delay treatment failed to follow the applicable standard of care by the defendant [4].

To properly analyze the standard of care question in the context of COVID-19, we must distinguish between crisis and noncrisis standards of care. In 2009, the Institute of Medicine published a landmark report stating that while noncrisis standards of care focus on the needs of each individual patient, crisis standards of care focus on public health emergencies. In other words, the standard of care physicians are expected to provide during the COVID-19 pandemic may, by necessity, be significantly different from standard nonemergency medical practice [6]. Therefore, this report that redefines the standard of care in a crisis setting, such as the pandemic, could be protective for healthcare providers during a time when extraneous circumstances impede their ability to provide the level of care expected in noncrisis times.

An empirically relevant set of guidelines was released by the American College of Surgeons (ACS) for the management of elective cancer surgery cases during the COVID-19 pandemic. The document provided a framework for physicians to consider various challenges of cancer patients’ needs, specifically during the pandemic. ACS stated that while most providers would not consider cancer surgery as elective, there is a hierarchy of urgency between the various surgical cases [7]. During the acute phase of the pandemic, subdivided into semi-urgent, urgent, and no ICU capacity, the guidelines recommend that surgeries should be restricted to patients without likely survivorship if not performed within the next three months, a few days, and a few hours, respectively, for each subdivision [7]. These recommendations, from a group of experts in the field of medicine, stratify the necessity for surgery based on the length of survival without it, aiding in defining which surgeries are deemed “elective.” Therefore, per the guidelines released by the ACS, the patient in this case would not have fallen into the category necessitating surgery during the acute phase of the pandemic.

Existing literature on this subject also discusses the lack of standardization in process, outcomes, and litigation when it comes to managing surgical patients during COVID-19. There was a request for a change in national policy to reflect the constraints placed on surgeons during an acute health crisis and for clear guidelines on protection for healthcare workers [8]. Without a set standard, it was recommended that surgeons use a decision-making framework that incorporates their obligations to patient-specific scenarios, resource stewardship, and patient advocacy [8]. Eventually, on April 14, 2020, the New Jersey Legislature passed P.L. 2020, c.18, which provided immunity to healthcare professionals in specific situations such as utilizing telemedicine, treating patients outside of one’s scope of practice, and the allocation of resources [9]. This was a temporary bill that served to provide immunity during the COVID-19 emergency and was later rescinded after the crisis was de-escalated in October of 2020.

## Conclusions

This case illustrates how state-mandated restrictions during the COVID-19 pandemic directly influenced treatment timelines for SCC. The Institute of Medicine and ACS guidelines provided a framework that supported surgical decision-making under these circumstances. Although the patient’s disease progressed, diligent care by the Otolaryngology Department led to an optimal outcome. While no litigation occurred in this scenario, the case highlights the intersection of crisis standards and medical-legal considerations.
